# Advances in Algin and Alginate-Hybrid Materials for Drug Delivery and Tissue Engineering

**DOI:** 10.3390/md21010014

**Published:** 2022-12-24

**Authors:** Qing He, Tianjian Tong, Chenxu Yu, Qun Wang

**Affiliations:** 1 Department of Mechanical Engineering, Tufts University, Medford, MA 02155, USA; 2 Department of Agricultural and Biosystems Engineering, Iowa State University, Ames, IA 50011, USA; 3 Department of Chemical and Biological Engineering, Iowa State University, Ames, IA 50011, USA

**Keywords:** algin/alginate, alginate hybrid materials (AHMs), drug delivery, tissue engineering

## Abstract

In this review, we aim to provide a summary of recent research advancements and applications of algin (i.e., alginic acid) and alginate-hybrid materials (AHMs) in medical fields. Algin/alginate are abundant natural products that are chemically inert and biocompatible, and they have superior gelation properties, good mechanical strengths, and biodegradability. The AHMs have been widely applied in wound dressing, cell culture, tissue engineering, and drug delivery. However, medical applications in different fields require different properties in the AHMs. The drug delivery application requires AHMs to provide optimal drug loading, controlled and targeted drug-releasing, and/or visually guided drug delivery. AHMs for wound dressing application need to have improved mechanical properties, hydrophilicity, cell adhesion, and antibacterial properties. AHMs for tissue engineering need improved mechanical properties that match the target organs, superior cell affinity, and cell loading capacity. Various methods to produce AHMs that meet different needs were summarized. Formulations to form AHMs with improved stability, drug/cell-loading capacity, cell adhesion, and mechanical properties are active research areas. This review serves as a road map to provide insights into the strategies to develop AHMs in medical applications.

## 1. Introduction

Alginic acid, also known as algin, is an edible polysaccharide in brown algae such as kelp, gulfweed, *Ascophyllum nodosum*, etc. It can also be produced through microbial fermentation [1]. Alginic acid is a polyanionic polysaccharide without branches. Alginic acid consists of repeating units of β-D-mannuronic acid (M) and units of α-L-guluronic acid (G) linked by a 1→4 linkage [2]. Its salts are known as alginates. Alginates can form hydrogel through crosslinking via divalent ions (Ca^2+^, Zn^2+^ and Cu^2+^) bridges [3]. Algin/alginates are used in a wide range of applications: catalysts [4,5], health care [6,7,8], water treatment [9,10,11], packaging [12,13,14], food additives [15], and cell culturing [16,17,18]. Their application in medical fields is snowballing in recent years due to their excellent biodegradability, biocompatibility, and non-toxicity [19]. 

Alginates are easy to handle and versatile; they can be modified chemically to develop drug and vaccine carriers [20]. Various alginate-based drug delivery systems have been developed, such as hydrogels [20,21], microparticles [19,22], nanoparticles [23], porous scaffolds [24], etc. Alginates can be further utilized in alginate-hybrid material (AHM) systems to improve drug loading rate, capacity, release control, and multi-response delivery [19,20,21,22]. In addition, along with targeted drug delivery, AHMs are also used in biosensing and imaging applications [22]. 

Moreover, algin/alginates and AHM are used in tissue engineering due to their gelation abilities through ion-induced crosslinking. The alginate-hydrogels display optimal mechanical strengths and biodegradability, which are crucial in tissue engineering as scaffolds. The AHMs developed by combining alginates with multifunctional materials such as skin fibroin, hyaluronic acid, gelatin, and polyvinyl alcohol were shown to have enhanced antibacterial properties, hydrophilicity, mechanical properties, and cell adhesion ability [25]. In addition, 3-D bioprinting with alginates-based bio-ink presented new opportunities in tissue engineering. The AHM-based hydrogel carriers can encapsulate bioactive cells and molecules to be printed directly to the damaged tissue [26] for in vivo or in vitro tissue repair. The AHM based on alginate-gum polymers enriched with TiO_2_ and SiO_2_ was developed to encapsulate the bioactive bacterium *Bacillus velezensis*. The innovative micro-encapsulation systems showed high encapsulation efficiency and could produce bioactive ingredients such as protease, lipase, siderophore, and auxin, promoting mineral phosphate dissolution [27]. This paper aims to review the most recent development in algin/alginate and AHMs in systems such as nanoparticles, microparticles, hydrogels, and scaffolds and their applications in drug delivery and tissue engineering. 

## 2. Alginate and AHMs in Drug Delivery

Alginate polymers have many attractive properties for developing drug delivery platforms, which include optimal biocompatibility, abundance in nature and low cost to produce, easily adaptable, chemically inert, superior gelation properties, etc [28]. They have been utilized in various drug delivery applications, especially in various AHMs. 

### 2.1. Targeted Drug Delivery with AHMs

Chitosan and alginate are marine-derived polysaccharides that are abundant and versatile, and both have shown excellent biocompatibility, biodegradability, and mucosal adhesion properties [19]. Chitosan-alginate hybrid (AH) nanoparticles have been developed as oral drug carriers for anti-cancer therapy. The electrostatic interactions between the amino groups of chitosan and the carboxyl groups of alginate are strong enough to facilitate the formation of stable AHM. AHM has excellent loading capacity for various bioactive compounds to protect them from the gastric environment. Gamboa et al. developed the chitosan-AH nanocomposite via spray freeze-drying as carriers loaded with prednisolone and inulin for targeted colonic delivery [23]. Chai et al. developed doxorubicin (DOX, a cancer medicine) loaded poly (lactic-co-glycolic acid) nanoparticles with chitosan-AH coating through a layer-by-layer assembly method; the formed nanocomplexes showed superior anti-sarcoma-tumor performance with only faint toxicity in vitro [29]. Martino et al. prepared folic acid (FA)-chitosan-AH nanocomplexes for the co-delivery of temozolomide and DOX [30], in which the addition of FA to the nanocomplexes improved their selective uptake by tumor cells. In vitro assay showed that the FA-chitosan-AH conjugates improved cell viability in HeLa cells but did not change the cell viability in NIH/3T3 cells. Rosch et al. prepared DOX-loaded chitosan-AH nanoparticles through a water-in-oil emulsification technique. The formed nanoparticles showed rapid uptake by 4T1 murine breast cancer cells in vitro [31]. 

**Figure 1:**
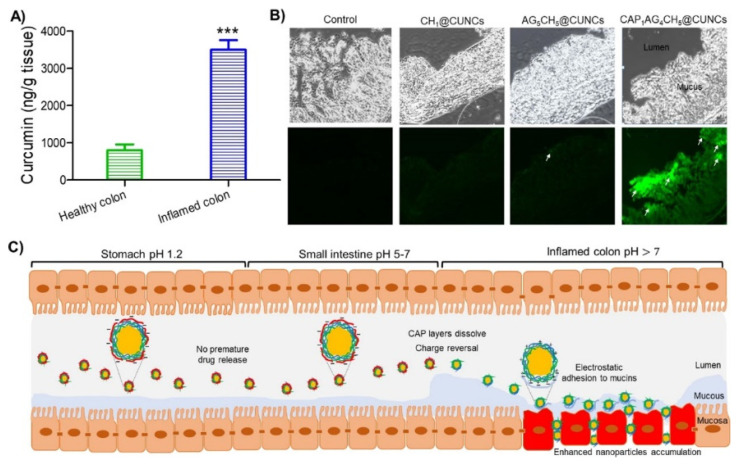
CH-coated CUNCs (CH1@CUNCs) and chitosan (CH), sodium alginate (AG) alternatively coated core−shell nanoparticles (AG4CH5@CUNCs) are accumulated in the inflamed colon in vivo. (**A**) Quantitative analysis of curcumin accumulated in the colon samples after oral administration of cellulose acetate phthalate (CAP) multilayer core−shell nanoparticles (CAP1AG4CH5@ CUNCs). (**B**) Confocal images showing the accumulation of CH1@CUNCs and core−shell nanoparticles in inflamed colons in mice. The core−shell nanoparticles are shown in green with white arrows. (**C**) Schematic illustration showing the adhesion and accumulation of CAP1AG4CH5@CUNCs in mice after oral administration. Data are represented as means ± SD (*n* = 6, *** *p* < 0.001) and replicated from Oshi et al. [33] with permission.

Chitosan-AHM could form crosslinked hydrogel at low pH due to electrostatic interactions. Drugs embedded in the hydrogel structure are protected against the harsh gastric environment and digestion in the gastrointestinal tract. For drug delivery to the colon, the Chitosan--AHM approach offers significant advantages, it could increase drug availability at the destination (e.g., colon), which could lead to better drug absorption, higher therapeutic efficiency, and reduced oral dosage to be administered (hence reduced potential toxicity) [32]. Oshi et al. developed chitosan-AH nanocarriers (CANCs, shell) for curcumin nanocrystals (CUNCs, core) in a core–shell structure to treat dextran sodium sulfate (DSS) induced inflammation colon model in colitis mice [33]. The nanocarriers precisely delivered CUNCs to the colon, as shown in Figure 1. The surface charge inversion triggered by the pH of CANCs improved the adhesion and accumulation of CUNCs in the colon. 

**Figure 2:**
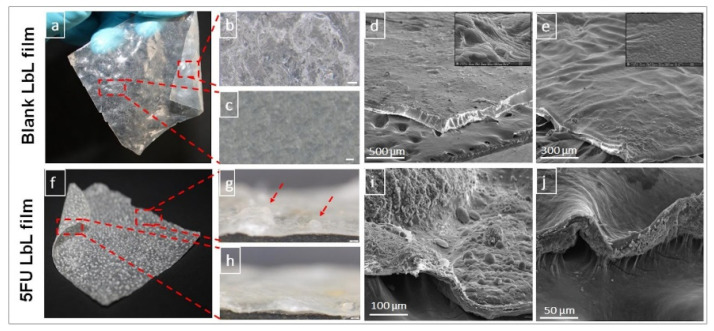
Photographs of blank layer-by-layer (LbL) film (**a**) and 5FU LbL film (**f**). Stereomicroscope images of PCL-side (**b**), chitosan-side (**c**), PCL-side (**g**), and chitosan-side (**h**) The scale bar (in **b** through **h**) represents 200 μm with arrows pointing to 5FU crystals protruding out-of-plane of LbL film. Scanning electron microscopic images of blank LbL film on the PCL-side (**d**) and chitosan-side (**e**) and 5FU LbL film on the PCL-side (**i**) and chitosan-side (**j**). Images are replicated from Janardhanam et al. [35] with permission.

Sarika et al. also developed cationic gelatin and sodium alginate polyelectrolyte complex nanoparticles for curcumin delivery [34]. The curcumin-loaded nanoparticles can be toxic to the cancer cell at 12.5 ug/mL concentrations. Janardhanam et al. developed a layer-by-layer (LbL) self-assembled chitosan-AH nanofilms (CANFs) coated with polycaprolactone (PCL) at the backside for oral delivery of 5-Fluorouracil (5FU) for colorectal cancer treatment (Figure 2) [35]. The LBL self-assembled films showed greatly improved loading efficiency for 5-FU. Moreover, the outer-most layer of the film is functionalized with folic acid to facilitate selective binding and targeting towards the tumor lesion. The 5-FU loaded CANFs showed higher stability and more robust growth inhibition against Caco-2 and COLO 320DM colorectal cancer cells than the 5-FU solution, demonstrating the potential of the CANFs as carriers for targeted delivery against colorectal cancer.

To prevent the burst release of medicines, AHMs were also produced with internal microvoids to achieve sustained release of drugs [36]. The drug-releasing profiles of hard delivery carriers (CaCO_3_), soft delivery carriers (alginate beads), and hard-and-soft CaCO_3_-AH systems were characterized, showing that the main factor controlling release from hard CaCO_3_ carriers was pH. In contrast, spontaneous diffusion controlled release from soft alginate carriers [37]. The CaCO_3_-AH system formed a stable slow-release drug delivery platform in which CaCO_3_ provided sufficient internal space and powerful attraction for the loading and storing drug molecules [38]. The CaCO_3_-AH carriers were shown capable of targeted drug delivery with sustained release. Zhao et al. developed CaCO_3_-AH nanocarriers loaded with DOX and DNA plasmids [39]; the nanocarriers showed high encapsulation efficiency and enhanced inhibition rate (i.e., 80%) against cell growth in HeLa cells. Compared with the CaCO_3_ carriers without alginate coating, the CaCO_3_-AHM system improved the delivery efficiency significantly.

Moreover, via acid etching of the CaCO_3_ core, the AHM carriers can be further transformed into porous or hollow shells that could perform even better as carriers. Boi et al. developed DOX-loaded alginate microbeads [36] by coating DOX-loaded CaCO_3_-AH microparticles alternatively with six layers of anionic polysaccharide dextran (DEX) and cationic polypeptide poly-arginine (PARG) obtain a (DEX/PARG)_3_ shell. The formed microbeads showed a mean diameter of 50 ± 9 μm and 37 ± 3%, respectively. The layers effectively limited drug leakage and controlled the initial burst release. The hybrid microbeads were also shown to provide effective release control in an assay with MCF-7 cells. In general, AHMs were shown to effectively control the slow release of drugs to improve their efficacy. They offer excellent platforms for drug delivery systems, although loading capacity and release control in these platforms could be further improved.

AHMs were investigated in various drug carrier scenarios for targeted cancer drug delivery [23]. Liu et al. developed a multi-response drug delivery nanogel with AHM of alginate and a thermosensitive polymer, poly(N-isopropyl acrylamide) (PNIPAM) [21]. The nanogel cross-linked alginate and PNIPAM in situ with cystamine as the bridging agent. The AH nanogel collapsed at 37 °C, then internalized by CAL-72 cells, and later swelled abruptly at 25 °C. Such thermo-responsive swelling behavior may induce cell death in tumor cells. Moreover, the AH nanogel exhibited accelerated release of DOX under acidic and reducing conditions, specific microenvironments inside solid tumors and endolysosomal compartments. Wang et al. developed acid-degradable collagenase-AH nanogels to enhance tumor penetration by polymerizing methacrylate alginate with ortho ester-containing monomers and subsequent immobilization of collagenase onto the AH nanogels [40]. Prabha and Raj et al. developed sodium alginate (SA)–polyvinyl alcohol (PVA)–bovine serum albumin (BSA) coated Fe_3_O_4_ nanoparticles (Fe_3_O_4_-SA-PVA-BSA) for delivering DOX [41]. The DOX-loaded nanoparticles demonstrated faster drug release in acidic conditions than in primary conditions. The DOX-loaded nanoparticles were shown to be able to kill HepG2 cells but were nontoxic to L02 cells. The effectiveness of these targeted delivery systems needs to be further validated via in vivo testing.

### 2.2. Multi-Response Delivery Schemes with AHMs

**Figure 3:**
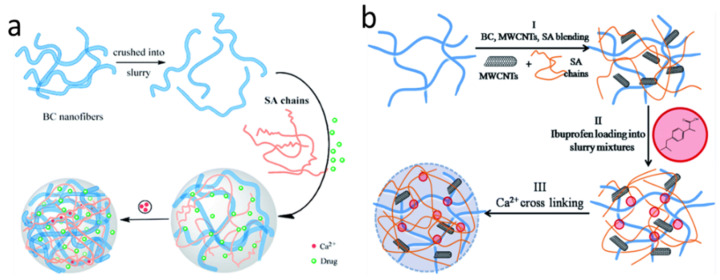
(**a**). The formation process for preparing the drug-loaded bacterial cellulose nanofibers (nf-BC) and sodium alginate (SA) (nf-BC-SA) hybrid hydrogels with a semi-interpenetrating polymer network (IPN) structure. (**b**). The schematic of the preparation of the drug-loaded sodium alginate (SA), bacterial cellulose (BC) and multi-walled carbon nanotubes (MWCNTs) (BC-SA-MWCNTs) hybrid hydrogels. Images are replicated from shi et al. [44,45] with permission.

AHMs can be produced to be responsive toward triggering chemical and electrical signals, which adds to their appeals as delivery systems. Muhammad Suhail et el. developed an acrylic acid-algin AH hydrogel as a sustainable drug delivery system for ketorolac tromethamine, which is responsive to pH changes [42]. The pH-responsive property of alginic acid enabled the controlled release of drugs in targeted microenvironments based on changes in acid-base balance, giving rise to dually controlled drug release. Cai et al. prepared nanocomplexes based on alginic acid-poly[(2-dimethylamino) ethyl methacrylate] (PDEMA) as delivery vehicles [43]. The alginic acid/PDEMA complexes were designed for ionic strength (via PDEMA) and pH (via algin)-controlled release of a model anticancer drug (DOX). Ions in a microenvironment could enter the nanocomplexes and destabilize them to create channels for drug release by shielding the charges of the polyelectrolytes. Shi et al. also created pH- and electro-response hydrogels based on bacterial cellulose nanofiber-sodium alginate (nf-BC-SA) (Figure 3a) [44]. The electrically stimulated (i.e., by introducing electrical current) release of drugs from the nf-BC-SA hybrid hydrogels was caused by the neutralization of H^+^ ions in the hydrogels (by electrons) leading to COO^-^ ionization and triggering swelling of the hydrogel matrix. They were leading to drug release as transport venues/channels were produced. Shi et al. further enhanced the electrical responses by adding a multi-walled carbon nanotube as the active ingredient (Figure 3b) to produce the bacterial cellulose-sodium alginate-multi-walled carbon nanotube hybrid hydrogel [45]. This hybrid material showed pulsatile release behaviors under repeated electrical pulsatile stimulation. Ge et al. also developed a Polypyrrole/Alginate Hybrid for electro- and pH-responsive dual-controlled drug delivery [46]. The external electric field generated a more potent electrostatic force which drove drugs through the hybrid hydrogel matrix and led to accelerated drug release. 

### 2.3. Multi-Functional Drug Carriers with AHMs

Dual- or multi-functional AHMs were developed for imaging and drug-delivery applications. AHMs with metal nanoparticles are developed as optical imaging contrast reagents. For example, Ahn et al. reported the development of g-Poly (N-isopropyl acrylamide) (PNIPAM)-AH micelles loaded with doxorubicin for targeted cancer drug delivery [47]. In another effort, radio-metal cross-linked AHM was developed for drug delivery and biomedical imaging with positron emission tomography (PET) and single photon emission computed tomography (SPECT) [48]. The radio-metal In^3+^ and Zr^4+^ were used as the contrast reagents for SPECT and PET, respectively. The radio-metal AH hydrogel showed great potential in cardiac tissue engineering, stem cell implantation, muscle implantation, oral drug delivery, and nasal drug delivery. Moreover, compared with other alginate imaging techniques with optical, fluorescence, MRI, CT, and ultrasound, SPECT/PET approach did not suffer from ambiguity of detection [48]. Core-shell-like AH microbubbles were developed by encapsulating shikonin (SHK) and indocyanine green (ICG) in alginate microbubbles. The developed SHK-ICG AH microbubbles served as dual-function material for drug delivery and ultrasound imaging enhancement (2.5 fold brightness increase achieved compared to CaCl_2_ medium) [49]. AH microspheres were developed based on the emulsification cross-linking of gelatin, genipin, and sodium alginate with low Curie temperature superparamagnetic iron oxide nanoparticles to improve the hyperthermia temperature control and drug delivery for transcatheter arterial chemoembolization (TACE). The developed microspheres can self-regulate the hyperthermia temperature at around 50 °C upon the alternating magnetic field, un-necessitating any temperature control facilities. Moreover, the embolic microspheres are detectable to CT/MR for monitoring [50]. The combination of drug delivery capabilities and imaging agents for real-time monitoring of how cells/tissues respond to treatment renders AHM systems multifunctional. Developing more of these AHM systems could add precision medicine tools.

## 3. Tissue Engineering with AHMs

Tissue engineering was developed to provide an alternative to organ transplant, which faces the everlasting challenges of organ donor shortage [51]. With the growing global population and life expectancy, the incidences of patients with diseased and injured tissues and organ failure are rising [52]. To address these needs, new materials have been investigated to make scaffolds that can be applied ex vivo or implanted in vivo to aid the regenerative processes of tissues and organs. Natural polymers such as hyaluronic acid [53], chitosan [54], gelatin [55], and alginate [56], or synthetic polymers such as poly (acrylic acid) or poly(lactide-co-glycolide) [57] have been used to produce hydrogel-based scaffolds. Among these materials, alginate and AHMs showed significant advantages due to their easily-implementable ion-triggered crosslinking mechanism to form a hydrogel. The alginate-based hydrogels had optimal mechanical strength and biodegradability, which are crucial for producing suitable scaffolds in tissue engineering applications. The cell attachment of alginate-based hydrogels can be further improved by utilizing AHMs [58,59]. 

### 3.1. Wound Dressing with AHMs

The skin is the “biggest” organ of our body. The human skin regulates the body temperature and protects us from mechanical injuries, microorganisms, toxins, and radiation from our surroundings [60]. Treatments to severe or chronic skin wounds, such as intense burn wounds, usually require a long time, which runs the risk of infections and is always a big challenge to manage [61]. Hence, advanced wound dressing that can minimize infection risk and accelerate healing is highly desirable. AHMs as a wound dressing has many advantages: they can prevent the entry of pathogenic bacteria more effectively, provide a moist environment and allow the wound exudates to be quickly evaporated, beneficiary towards healing. Some other multifunctional materials are combined with alginate and its derivatives to make AHMs with improved properties, such as hydrophilicity, mechanical properties, and cell adhesion abilities [25]. Ghalei et al. prepared alginate hydrogel-electrospun silk fibroin fibers for amniotic fluid delivery to wounds [62]. The AHM improved the amniotic fluid delivery, resulting in better cell proliferation, spreading, and collagen formation, which helped promote wound healing. Abou-Okeil et al. developed a topical bioactive hyaluronic acid/sodium alginate film crosslinked with cations (Ca^2+^, Zn^2+^ and Cu^2+^) for wound dressing [3]. The results confirmed that the formed AHM with sulfadiazine and silver nanoparticles had good homeostasis properties. 

### 3.2. Antibacterial Wound Dressing with AHMs

**Figure 4:**
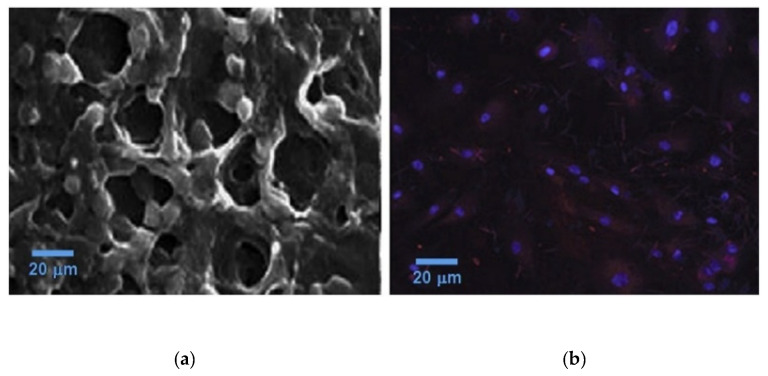
(**a**) The SEM and (**b**) the confocal microscopic images of osteoblast cells cultured on silver hydroxyapatite-reinforced gelatin-alginate-PVA scaffolds. Images are replicated from Kumar Saini et al. [70] with permission.

**Figure 5:**
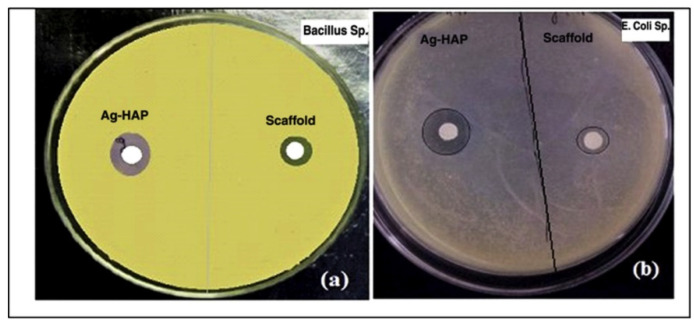
Antibacterial study of silver hydroxyapatite-reinforced gelatin-alginate-PVA scaffolds (**a**) Bacillus sp. and (**b**) E. coli sp., respectively. Images are replicated from Kumar Saini et al. [70] with permission.

Algin and its derivatives have been investigated to produce antibacterial AHMs for wound dressing and other applications. Silver nanoparticles (AgNP) were shown to have practical antimicrobial effects [63,64,65]. They could release Ag+ ions to interact with bacterial cell walls and membranes to trigger cell death. Moreover, the Ag+ can bind with the thiol (R-SH) group of membrane proteins to inhibit cell respiration [65,66,67]. They could be particularly effective against antibiotic-resistant bacteria. However, AgNPs are cytotoxic, which limits their applications. Algin and its derivatives were utilized to produce AgNP-AHMs for antibacterial applications targeting antibiotic-resistant bacteria. Belattmania et al. extracted sodium alginate from *Sargassum muticum* to synthesize AgNP-AHM [68]. The sodium alginate functions as the reducing and stabilizing agent for the AgNPs. This hybrid material showed antibacterial activity against various bacteria. Zhang et al. developed AgNP-collagen-AHM, which showed cytotoxicity on mouse embryonic fibroblasts (NIH3T3) and antibacterial activity against *E. coli* and *S. aureus* [69]. Kumar Saini et al. developed silver-hydroxyapatite loaded gelatin- PVA-AH cryogels which formed a porous scaffold (Figure 4). The scaffolds showed antibacterial activities against *E. coli* and *B. subtilis* (Figure 5) [70] which could be used as antibacterial tissue engineering platform. 

Carbon nanomaterials such as graphene and its derivatives showed negligible mammalian cytotoxicity yet good antibacterial properties and mechanical properties. Therefore, they have been used to develop AHMs for antibacterial applications. Miguel Martí et al. developed the calcium alginate-graphene oxidate AHM to form a film with reinforced mechanical properties and solid antibacterial activities against *Staphylococcus aureus* and methicillin-resistant *Staphylococcus epidermidis*, yet low cytotoxicity towards human keratinocyte HaCaT cells [71].

### 3.3. Bone Tissue Engineering with AHMs

Bone is the most transplanted tissue. Until now, no fully functional bone equivalents that allow vascularization and integration have been developed to replace the complex human bone tissue [26,72]. An optimal bone substitute must be a matrix for osteogenic cells and osteoinductive factors and provide sufficient mechanical strength to promote vascularization [73]. Hydrogels are the mainstay of scaffold material for tissue engineering due to their softness and porous structure that allows nutrients and oxygen diffusion [74,75]. Alginate hydrogel and AMH hydrogels are the most studied tissue engineering materials as they provide an effective matrix for cell loading. AHM can be injected directly into the damaged area with encapsulated bioactive cells and molecules, and then ionic-induced crosslinking can be triggered to start the bone repair process [26]. Ansari et al. developed hyaluronic acid-AH hydrogel loaded with transforming growth factor beta 1 (TGF-β1) as a stem cell delivery system [76], the formed hydrogel encapsulated with periodontal ligament stem cells (PDLSCs) provided a continued release of TGF-β1 and chondrogenic differentiation up to 14 days. Segredo-Morales et al. prepared a thermos-responsive poloxamine-AH hydrogel injectable to regenerate osteoporotic bone defects [77]. This system exhibited quick release of 17β-estradiol (βE) / bone morphogenetic protein (BMP)-2 in the first three days, followed by the second phase of long and slow release. In vivo evaluation in rats showed a synergistic effect between βE and BMP-2, which yielded a higher percentage of drug release.

Yan et al. developed bacterial cellulose nanocrystal (BCN)-AH scaffolds by internal gelation [78]. The formed hydrogel scaffolds showed porous microstructures and desired mechanical and biological activities. The layer-by-layer successive electrostatic assembly facilitated biodegradative behavior and cell attachment. Incorporating BCN in the AH hydrogel improved the compressive strength and biodegradability, increasing the scaffold’s porosity and favoring MC3T3-E1 cells. Developing the AHM carriers for delivering cells and bone growth factors is the essential application of AHM in bone tissue engineering. Alginate alone lacks the cell adhesive and mechanical properties for such applications. Moreover, to facilitate bone tissue regeneration, the growth factors need to be released in a controlled manner [73]. Thereby, AHMs need to be developed with properties tailor-optimized for specific applications. 

### 3.4. Cartilage Tissue Engineering with AHM

**Figure 6:**
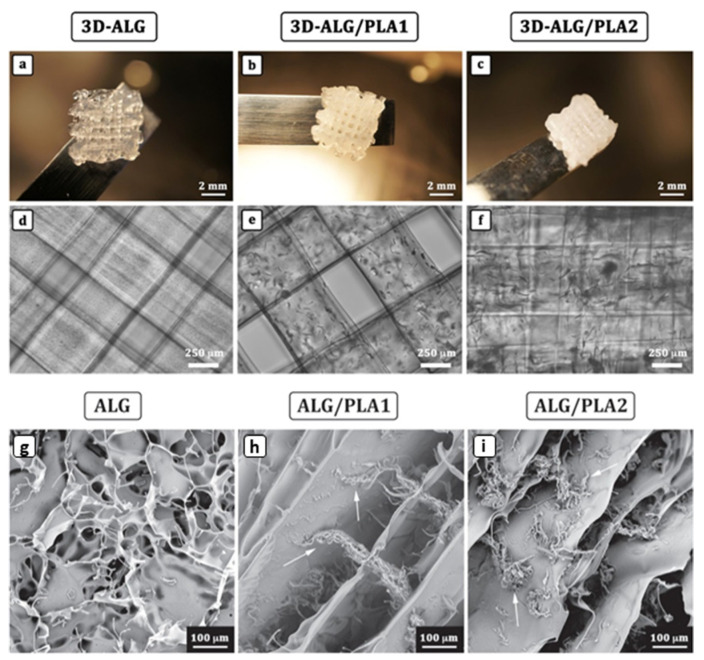
The 3D-printed scaffolds by alternate layers of hydrogel filaments oriented 0°–90°. (**a**) 3D-ALG −3.5 wt% alginate, (**b**) 3D-ALG/PLA1 −3.5 wt% alginate + 1% PLA, and (**c**) 3D-ALG/PLA2 −2.5 wt% alginate + 2% PLA. Overall dimensions of the printed scaffolds: 7 × 7 × 2 mm. Images are replicated from Kosik et al. The flow curves of the different formulations find a direct evidence from the analysis of the optical micrographs of the deposited strands (**d**) 3D-ALG, (**e**) 3D-ALG/PLA1, (**f**) 3D-ALG/PLA2. SEM of dried bioink solutions w/o the addition of short sub-micron PLA fibers: (**g**) ALG (3.5 wt% alginates), (**h**) ALG/PLA1 −3.5 wt% alginate + 1% PLA and (**i**) ALG/PLA2 −2.5 wt% alginate + 2% PLA. Adapted from [84] with permission.

The cartilage minimizes or eliminates friction due to load transference and movement of joints. Cartilage forfeiture can be caused by injuries or diseases, which, if unable to self-heal, pose a challenge to medical repair due to the functional and structural complexity of the avascularity of the cartilage [79,80]. Both in vivo and in vitro research have shown that cartilage damage can be artificially repaired by inserting alginate/AH scaffolds [81,82]. Olubamiji et al. developed a PCL-AH 3D printed cartilage construct loaded with mouse chondrogenic cell line ATDC5 for cartilage repair [83]. The in vivo experiments showed a delineated cell viability of ∼73% when implanted for 21 days, confirming cartilage tissue regeneration. Kosik-Kozioł et al. developed short sub-micron polylactide (PLA)-AH 3D-printed hydrogel constructs loaded with human chondrocytes for cartilage tissue engineering (as shown in Figure 6) [84]. The addition of PLCA short fibers increased Young’s modulus of the AHM construct threefold up to 25.1 ± 3.8 kPa. In the in vitro test, the construct was cultured with human chondrocytes for up to 14 days, with the cells showing a high retention rate, viability (∼80%) and a healthy, round morphology. Kreller et al. developed a 3D-printed alginate di-aldehyde-gelatine (ADA-GEL) hydrogel to create hierarchically ordered scaffolds for cartilage tissue engineering [55]. ADA-GEL mimics the intrinsic hierarchical structure of natural cartilage tissue. The formed scaffold could host chondrocytes to support rapid healing for cartilage tissues. The key to developing AHMs for cartilage tissue engineering is to improve the mechanical properties to allow the material to stay in the load-bearing site without degradation. At the same time, the cells undergo cartilage tissue regeneration [85]. This could be realized by developing the AHMs with improved cell loading and affinity, mechanical properties, and different formations such as scaffolds and fibers. 

### 3.5. Brain Tissue Engineering with AHMs

Cell transportation and tissue engineering have been extensively studied as a treatment for neurological disorders. The proper materials used are crucial to cell transportation and tissue engineering success. AHMs have been explored as carriers and matrixes for cell delivery and tissue engineering for brain and neurological disorder therapies due to their natural origin, biocompatibility, and easy manipulation. Li et al. used gelatin-AHM composite as the base hydrogel loaded with Schwann cells RSC96s for brain tissue engineering [18]. Due to the faster release of neuron growth factors in the 3D scaffold, they reported ∼92.34% viability in cells after 14 days of culturing in a 3D printed scaffold, compared to ∼85.35% viability in cells with a 2D scaffold. The alginate-based hybrid hydrogels bio-scaffolds is developed due to its controllable properties that could facilitate accurate cell delivery, cell survival and differentiation, and controlled release of stem cells to the target site. The developed bio-scaffolds are injectable via needles and detectable through manganese-enhanced MRI when capsulated with Mn ions or Mn nanoparticles. In vitro tests proved that human adipose-derived stem cells could survive in the developed AH hydrogels for at least 14 days [86]. The Mn^2+^ alginate hydrogel AHM was developed to improve the precision and efficacy of cell therapy for central nervous system disorders. The developed AHM showed improved cell viability and a lack of immune response. The Mn^2+^-labeled AHM produced a strong T1 MRI signal [87]. The AHM also showed its potential in neuro-regenerative applications. An electric field could be applied to stimulate the resident brain neural precursor cells (NPCs) to proliferate and differentiate, facilitating neural repair. The injectable alginate and Poly(3,4-ethylene dioxythiophene) AHM was developed as a biocompatible soft electrode for in vivo electrical stimulation for NPCs. The AHM showed mechanical properties (i.e., modulus) that matched with the brain tissues, hence was suitable for injection, and caused minimal inflammatory responses upon injection [88]. The chitosan on alginate-based macroporous hydrogels AHM was also developed for brain cancer capture in the surgical cavity following cancer cell removal after stereotactic radiotherapy. The developed AHM showed a good compromise between low proliferation and high accumulation of F98 with optimal hydrogel formula [89]. AHMs showed the potential to be an ideal matrix material for cell transportation and tissue engineering for treating neurological disorders, which should be further explored in future work.

### 3.6. Tissue Engineering with AHMs in Organs

**Figure 7:**
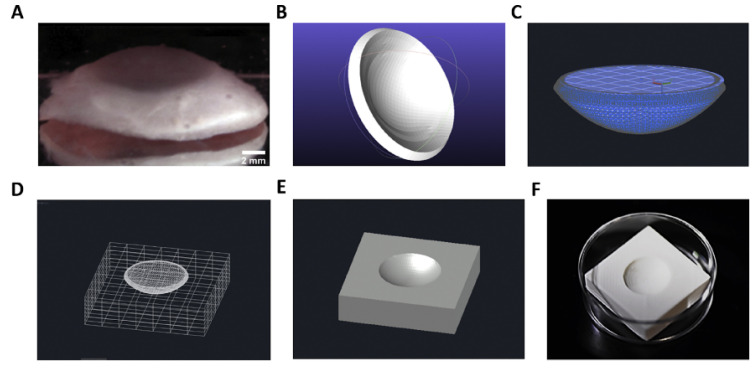
Stages support structure generation. (**A**) The human cornea shows the size and natural curvature across the surface. (**B**) The original corneal model was derived by (Simonini and Pandolfi, 2015) via the Finite Element Method (FEM). The cornea is then ‘sealed’ (**C**) with a planar circle to subtract its volume from the support structure. (**D**) Wireframe view of cornea situated at the center of cuboid before subtraction. (**E**) Digital support structure after subtraction. (**F**) The 3D printed plastic support structure. Images are replicated from Isaacson et al. [90] with permission.

AHMs have also been developed for cell delivery and tissue construction for organs. Isaacson et al. modified the corneal curvature of the eye by developing a collagen-AHM to cultivate keratocytes for implanting in the eye (Figure 7) [90]. Crosslinked alginate by calcium bestowed stiffness and transparency upon the scaffold. The mechanical properties of the collagen-AHM could be tuned by adjusting the collagen concentration. Moreover, the collagen-AHM conferred 83% cell viability for keratocytes at day 7 of incubation. Hiller and Berg et al. developed a bio-ink composed of gelatin-AHM loaded with human HepaRG liver cells, which was then printed with a pneumatic extrusion printer for liver tissue engineering [16]. The printed gelatin-AHM constructs showed good stability and supported good viability and metabolic functions for the embedded HepaRG cells. The resulting mini-liver model to study the infection caused by human adenovirus 5 (hAdV5). The results showed that mini-liver supported efficient adenoviral replication, which is suitable for studying viral biology and screening for new antiviral compounds. 

## 4. Conclusions

In this review, we summarized the recent development and application of AHMs in medical applications. Algin and alginates showed excellent biocompatibility and versatility. They are chemically manipulable, easy to acquire at low cost and form hydrogel with simple ion-induced crosslinking with good mechanical strength, and biodegradable. Various materials, from natural and synthetic polymers, to inorganic nanomaterials, can be utilized to form AHMs with improved stability, drug loading rate/capacity, cell adhesion, mechanical strength and properties for their specific applications. These AHMs were excellent candidates for drug delivery carriers, wound dressing, cell culture and delivery matrix for tissue engineering, etc. In the drug delivery application, many endeavors have been made to develop the AHMs with improved targeted drug delivery abilities, higher drug loading, optimized drug releasing profile, and controlled drug delivery. Moreover, recent developments have been focused on muti-function AHMs for drug delivery and imaging. The AHMs with targeted drug delivery, controlled drug release and imaging functions can be beneficial for developing precision medicine. Future work in this field needs to focus on properly evaluating the effectiveness of AHM-based systems in vivo as well as in clinical trials, to truly take advantage of the improved properties and mulri-functionality in these hybrid systems. 

The AHMs also showed great potential in the tissue engineering field; the recently developed AHMs showed improved mechanical properties that match the targeted tissues and improved cell affinity and loading for faster tissue regeneration. Furthermore, some efforts have been made to develop the AHMs for creative applications such as cancer cell-targeted entrapping and in vivo electrical stimulation for NPCs differentiation. In general, AHMs with better cell loading and mechanical properties are still crucially needed for their applications in tissue engineering. Additionally, future research needs to go beyond lab testing stage and the AHM-based systems need to be tested in clinical trials. In addition, the efforts to develop AHMs for tissue engineering novel applications other than cell and molecule delivery are also needed. This review serves as a road map to provide insights into strategies to develop better AHMs for future medical applications.
